# Insight Into the Binding Mechanism of p53/pDIQ-MDMX/MDM2 With the Interaction Entropy Method

**DOI:** 10.3389/fchem.2019.00033

**Published:** 2019-01-29

**Authors:** Mengxin Li, Yalong Cong, Yuchen Li, Susu Zhong, Ran Wang, Hao Li, Lili Duan

**Affiliations:** ^1^School of Physics and Electronics, Shandong Normal University, Jinan, China; ^2^Department of Science and Technology, Shandong Normal University, Jinan, China

**Keywords:** polarized force field, interaction entropy, molecular dynamics simulations, binding free energy calculation, p53-MDMX/MDM2

## Abstract

The study of the p53-MDMX/MDM2 binding sites is a research hotspot for tumor drug design. The inhibition of p53-targeted MDMX/MDM2 has become an effective approach in anti-tumor drug development. In this paper, a theoretically rigorous and computationally accurate method, namely, the interaction entropy (IE) method, combined with the polarized protein-specific charge (PPC) force field, is used to explore the difference in the binding mechanism between p53-MDMX and p53-MDM2. The interaction of a 12mer peptide inhibitor (pDIQ), which is similar to p53 in structure, with MDMX/MDM2 is also studied. The results demonstrate that p53/pDIQ with MDM2 generates a stronger interaction than with MDMX. Compared to p53, pDIQ has larger binding free energies with MDMX and MDM2. According to the calculated binding free energies, the differences in the binding free energy among the four complexes that are obtained from the combination of PPC and IE are more consistent with the experimental values than with the results from the combination of the non-polarizable AMBER force field and IE. In addition, according to the decomposition of the binding free energy, the van der Waals (vdW) interactions are the main driving force for the binding of the four complexes. They are also the main source of the weaker binding affinity of p53/pDIQ-MDMX relative to p53/pDIQ-MDM2. Compared with p53-MDMX/MDM2, according to the analysis of the residue decomposition, the predicated total residue contributions are higher in pDIQ-MDMX/MDM2 than in p53-MDMX/MDM2, which explains why pDIQ has higher binding affinity than p53 with MDMX/MDM2. The current study provides theoretical guidance for understanding the binding mechanisms and designing a potent dual inhibitor that is targeted to MDMX/MDM2.

## Introduction

Protein-Protein interactions play an important role in the recognition of numerous biological processes and biomacromolecules (Pawson and Nash, 2000; Wang et al., 2001; Keskin et al., 2008). Investigating protein-protein interactions at the atomic level via MD simulation can yield quantitative information and is helpful for understanding the microscopic mechanisms of biological processes. Many vital biological processes, such as enzyme catalysis, gene expression, and adjustment of signal pathways (Schreiber and Fersht, 1995, 1996; Pazos et al., 1997), are inseparable from the adjustment of protein interactions (Schreiber et al., 2009), which are at the heart of many essential biological process. The design of drugs based on protein-protein interactions has been become a hot topic. Since the origins of many diseases are closely related to protein disorders, the design of drugs that are aimed at regulating the structure, and function of proteins are becoming a focus of researchers. The binding strength between two proteins is determined by the binding free energy. Thus, accurate calculation of the binding free energy is vital for investigating the interaction mechanism and is helpful for drug design. Drug-like molecules tend to bind to hot areas (Burgoyne and Jackson, 2006; Cheung et al., 2012) in protein-protein interaction surfaces.

Molecular dynamics (MD) simulation (McCammon et al., 1977) is an important tool for exploring the characteristics of biomacromolecules. The accuracy of a simulation is determined by the force field that is used. However, the popular force fields, such as CHARMM, AMBER, GROMOS, and OPLS, do not consider the electrostatic polarization effect; hence, they may yield inaccurate results (Gao et al., 2011). In this study, the polarized protein-specific (PPC) force field, which fully considers the effect of electrostatic polarization, is used in the MD simulation. PPC is developed by the Zhang group (Ji et al., 2008) and many studies have demonstrated its advantage over the traditional force fields (Ji and Zhang, 2008; Duan et al., 2010, 2016a; Ji and Mei, 2014).

The calculation of the binding free energy between two proteins is the key issue in computer simulation and drug design. Precise free energy prediction methods can substantially improve the efficiency of drug design. At present, there are rigorous approaches to calculate the binding free energy, such as free energy perturbation (FEP) (Rao et al., 1987; Cummins and Gready, 1993) and thermodynamics integration (TI) (Straatsma and Berendsen, 1988; Aqvist et al., 1994). Although these methods are highly accurate (Chen et al., 2017a) in the calculation of the binding free energy, they are time-consuming and computationally expensive. In addition, they can only compute the relative binding free energy. In contrast, the Molecular Mechanics/Poisson-Boltzmann Surface Area (MM/PBSA) (Srinivasan et al., 1998; Wang and Kollman, 2000; Wang et al., 2001) method has been widely used in the calculation of absolute binding free energy due to its high efficiency (Sun et al., 2014; Genheden and Ryde, 2015; Chen et al., 2016). However, a serious problem with the MM/PBSA method is that calculating the entropic contribution is difficult. Typically, the normal mode (Nmode) method is used to compute the entropy change (Ngyuen and Case, 1985; Xu et al., 2011); this method is inaccurate for the study of biomacromolecules (Wang et al., 2016). In this paper, a novel method, namely, interaction entropy (IE) (Duan et al., 2016b), is used to calculate the entropy change, which is theoretically rigorous. It can yield more accurate results than the traditional Nmode method, which has been applied successfully to the calculation of the binding free energy in many studies (Duan et al., 2016a, 2017a,b; Aldeghi et al., 2017; Ben-Shalom et al., 2017; Cebrián-Prats et al., 2017; Chen et al., 2017a,b; Gao et al., 2017; Khammari et al., 2017; Nguyen Quoc et al., 2017; Sun et al., 2017; Wang et al., 2017; Yan et al., 2017; Yang et al., 2017; Zarei et al., 2017; Zou et al., 2017; Cong et al., 2018; Liu et al., 2018; Qiu et al., 2018; Song et al., 2018).

Typically, residue decomposition is performed to obtain hot-spot residues in the MM/GBSA method (Martins et al., 2013; Simões et al., 2017). However, the entropy contribution cannot be computed via the Nmode method in the analysis of the residue decomposition, which results in overestimation of the predicted binding free energy of each residue. Fortunately, the IE method can calculate the entropy change of per residue, which overcomes this shortcoming of the traditional MM/GBSA method.

p53 is important tumor suppressor and transcription factor. It helps protect the integrity of the genome. The structures of oncoproteins MDMX and MDM2 are highly similar, which can inhibit the activation of p53. Overexpression of MDMX and MDM2 can cause function loss of p53, thereby resulting in the occurrence of cancer. According to numerous studies, more than 50% of malignant tumors are related to p53 (Vogelstein et al., 2000; Joerger and Fersht, 2007; Vazquez et al., 2008); hence, p53-MDMX/MDM2 interaction is an important target for tumor drugs. In many studies, drug design that is aimed at adjusting the p53 pathway is becoming popular. Chang et al. demonstrated that stapled α-helical peptide can be developed as an inhibitor of the interaction of p53 with MDMX and MDM2 (Chang et al., 2013). Tsuganezawa et al. developed a new method, namely, high-throughput screening (HTS) assay, for identifying drug inhibitors for the p53-MDMX interaction (Tsuganezawa et al., 2013). Verma et al. investigated the binding mechanism of a potential inhibitor, namely, polyphenol, to MDM2 via molecular docking and molecular dynamics simulation and found that luteolin has high inhibition potency for MDM2 (Verma et al., 2016). Few researchers have directly explored the interaction mechanisms between p53-MDMX and p53-MDM2, especially via the combination of PPC and IE methods. In addition, a peptide inhibitor, namely, pDIQ, that has high binding affinity for both MDMX and MDM2 was designed by Phan et al. (2010). for disrupting the interaction of p53-MDMX/MDM2 to maintain the activation of p53. In this paper, the binding mechanisms of p53-MDMX/MDM2 and pDIQ-MDMX/MDM2 are investigated via the combination of PPC and IE to obtain detailed binding information. The current work will provide important information for the design of dual-function inhibitors.

## Methods

### MD Simulation

In current study, the initial structure of p53-MDMX (3DAB), p53-MDM2 (1YCR), pDIQ-MDMX (3JZQ), and pDIQ-MDM2 (3JZS) are obtained from Protein Databank (PDB). The parameters of the proteins are generated from the AMBER12SB force field. The Leap module is used to add all missing hydrogen atoms automatically. The four systems are solvated in the truncated periodic octahedral box of TIP3P waters to provide solvent environment, in which, the distance between the surface of the complex and the edge of the periodic box wall is 10 Å. The counter ions are added to neutralize the systems. To remove the steric clashes, the systems are relaxed by energy minimization, which is performed by the steepest descent method, followed by conjugate gradient minimization. Then the whole systems are heated form 0 to 300 K up for 300 ps with the step of 2 fs. SHAKE (Ryckaert et al., 1977) algorithm is used to constrain all bonds involving hydrogen atoms. To prevent the unnecessary structural drift in protein-protein systems, the restrained MD simulations are performed up to 12 ns and 10 fs per frame is written to get enough conformational sampling from the last equilibrium stage for the four systems.

### Molecular Mechanics/Poisson-Boltzmann Surface Area

In this paper, the MM/PBSA model (Massova and Kollman, 2000) is adopted to calculate the binding free energy. The binding free energy can be expressed by the following terms:

(1) $$\begin{array}{l} \Delta G_{bind}=\;G_{complex}-\left(G_{protein1}+G_{protein2}\right) \end{array}$$

Where *G*_*complex*_, *G*_*protein*1_, *G*_*protein*2_ represent the free energy of complex, p53/pDIQ, MDM2/MDMX, respectively. Additionally, the binding free energy is expressed by the sum of the following two terms:

(2) $$\begin{array}{l} \Delta G_{bind}=\Delta G_{gas}+\Delta G_{sol} \end{array}$$

Where Δ*G*_*gas*_, Δ*G*_*sol*_ represent the gas phase binding free energy and solvation free energy, respectively. Δ*G*_*gas*_ is divided into three terms:

(3) $$\begin{array}{l} \Delta G_{gas}=\Delta E_{ele\;}+\Delta E_{vdW}-T\Delta S \end{array}$$

Δ*E*_*ele*_, Δ*E*_*vdW*_, and −*TΔS* represent electrostatic interaction, van der Waals (vdW) interaction and entropic contribution, respectively.

The *G*_*sol*_ terms can be expressed by the sum of the polar and non-polar solvation free energy. The formula is as follows:

(4) $$\begin{array}{l} \Delta G_{solv}=\Delta \;G_{pb}+\Delta \;G_{np} \end{array}$$

The first term of the formula is calculated by applying the PB equation. During the calculation, the internal, and external dielectric constants are set to 1 and 80, respectively. The second term Δ*G*_*np*_ can be calculated by the following formula:

(5) $$\begin{array}{l} \Delta G_{np}=\gamma .SASA+\beta \end{array}$$

*SASA* represents the solvent accessible surface area, which can be calculated by using the MSMS (Sanner et al., 1996) program. The values of γ and β are 0.00542 kcal (mol Å^2^)^−1^ and 0.92 kcal/mol, respectively. In our calculation, MM/PBSA method is performed based on 100 snapshots from MD simulation trajectory.

### Interaction Entropy Method

In this paper, a new developed Interaction Entropy (IE) (Duan et al., 2016b, 2017a) method is applied to calculate the entropy change. All snapshots extracted from the last equilibrium MD simulation are used to the calculation of entropic contribution. This can be obtained from the following formula. The gas-phase component of the binding free energy for the interaction of protein and protein can be expressed by the following equations:

(6) $$\begin{array}{l} \Delta G_{gas}=\;-KTln\left[\frac{Q_{p1p2}}{Q_{p1p2}^{\prime }}\right]\\ =-KTln\frac{\int dq_{w\left(p1p2\right)}dq_{p1}dq_{p2}e^{-\beta \left(E_{p1}+E_{p2}+E_{p1-p2}^{int}+E_{w\left(p1p2\right)}+E_{p1p2-w}^{int}\right)}}{\int dq_{w\left(p1p2\right)}dq_{p1}dq_{p2}e^{-\beta \left(E_{p1}+E_{p2}+E_{w\left(p1p2\right)}+E_{p1p2-w}^{int}\right)}}\\ =-KTln\left[\frac{1}{\langle e^{\beta E_{p1-p2}^{int}}\rangle }\right]\\ =KTln\langle e^{\beta E_{p1-p2}^{int}}\rangle \\ =\langle E_{p1-p2}^{int}\rangle +KTln\langle e^{\beta {\Delta E}_{p1-p2}^{int}}\rangle \\ =\langle E_{p1-p2}^{int}\rangle -T\Delta S \end{array}$$

So the interaction entropy can be defined as the following equation:

(7) $$\begin{array}{l} -T\Delta S=KT\ln\langle e^{\beta \Delta E^{int}}\rangle \end{array}$$

The Δ*E*^*int*^ is defined as the fluctuation of protein-protein interaction energy around the average energy. It can be expressed as the following equation:

(8) $$\begin{array}{l} \Delta E^{int}=E^{int}-\langle E^{int}\rangle \end{array}$$

The 〈*E*^*int*^〉 is averaged protein-protein interaction energy. This term of equation can be calculated by the formula:

(9) $$\begin{array}{l} \langle E^{int}\rangle =\frac{1}{N}\overset{N}{\underset{i=1}{\sum }}E^{int}\left(t_{i}\right) \end{array}$$

The 〈*e*^βΔ*E*^*int*^^〉 term of equation can be calculated by the formula:

(10) $$\begin{array}{l} \langle e^{\beta \Delta E^{int}}\rangle =\;\frac{1}{N}\;\overset{N}{\underset{i=1}{\sum }}e^{\beta \Delta E^{int}\left(t_{i}\right)} \end{array}$$

For the above formulas, β represents $\frac{1}{KT}$.

## Results and Discussion

### The Analysis of Stability

**Figure 1:**
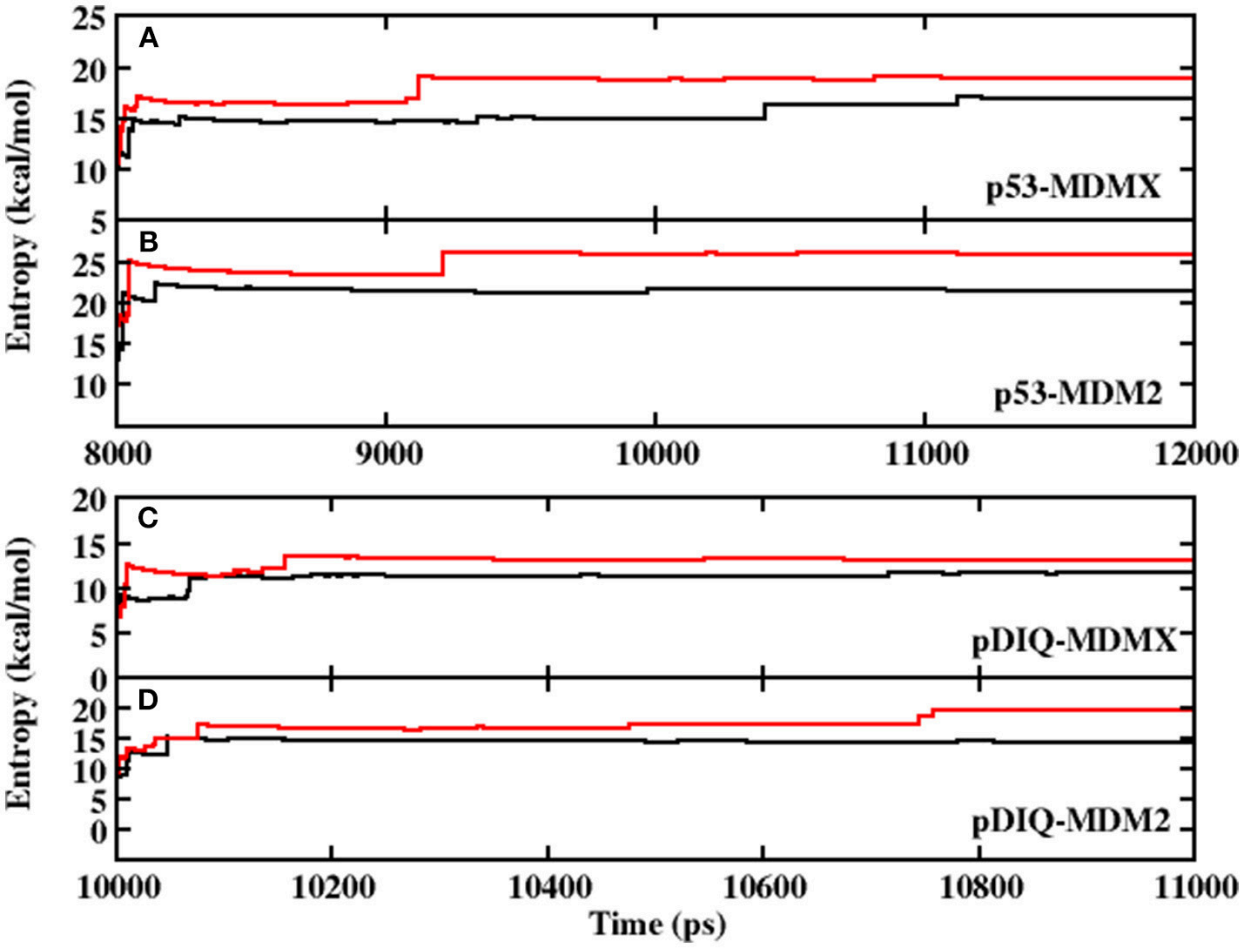
The calculated interaction entropy under AMBER and PPC as a function of equilibrium time fromMD simulation. **(A)** p53-MDMX system; **(B)** p53-MDM2 system; **(C)** pDIQ-MDMX; **(D)** pDIQ-MDM2.

Prior to analyzing the binding free energy, to ensure the stability of the protein in the dynamic simulation and the convergence of the calculated interaction entropy, the root-mean-square deviation (RMSD) of the backbone atoms relative to the native structure is shown in Figure S1 in Supporting Information. As shown in Figure S1, those structures of p53/pDIQ-MDMX/MDM2 are highly stable during MD simulation. Figures 1A,B show the interaction entropy as function of time in the 3DAB and 1YCR systems, respectively, where the black line represents the calculated results from AMBER and the red line represents the calculated results from PPC. The entropy change well converges in the MD simulation under the two force fields. In addition, the difference in the calculated entropy between AMBER and PPC is not large according to the figure; hence, the polarization effect has no substantial impact on the entropy change.

### The Analysis of Binding Free Energy

To obtain detailed information on the binding mechanisms of p53-MDMX and p53-MDM2, the binding free energies are calculated under two combinations (AMBER-IE, PPC-IE). The results are listed in Table 1, in which the experimental values of 3DAB and 1YCR are −9.12 and −9.20 kcal/mol, respectively. The difference between the two experimental values is −0.08 kcal/mol. Under PPC-IE, the calculated binding free energies of 3DAB and 1YCR are −27.44 and −28.93 kcal/mol, respectively. The difference between them is −1.49 kcal/mol, which accords with the difference between the experimental values. However, the calculated binding free energies of 3DAB and 1YCR under AMBER-IE are −29.12 and −26.95 kcal/mol, respectively. The difference between the calculated binding free energies is 2.17 kcal/mol, which differs substantially from the experimental result. Additionally, the calculated standard deviations (STDs) in Table 1 are all very low; hence, the results are reliable. The rank of the computed binding free energy is consistent with the rank of the experimental data under the PPC-IE method; therefore, PPC-IE is the superior choice and the following analysis will be based on the computations with PPC-IE. According to Table 1, the binding free energy of MDM2 with p53 is higher than that of MDMX with p53. To further explore the binding mechanisms of p53 and MDM2/MDMX, the contributions of the binding free energy components are listed in Table 2. The binding energy of p53-MDMX is larger than that of p53-MDM2. Moreover, the electrostatic interactions, vdW interactions, and non-polar solvation energies contribute favorably to the binding free energy, while the polar solvation energy and entropy change play unfavorable roles.

**Table 1:** Binding free energy for p53-MDMX/MDM2 and pDIQ-MDMX/MDM2 calculated by the combination of AMBER-IE and PPC-IE.

**System**	**AMBER**							**PPC**							ΔG_exp^*^_
	**$\langle {\text{E}}_{\text{pp}}^{\mathrm{int}}\rangle$**	**STD**	**−TΔS**	**STD**	**ΔG_sol_**	**STD**	**ΔG_bind_**	**$\langle {\text{E}}_{\text{pp}}^{\mathrm{int}}\rangle$**	**STD**	**−TΔS**	**STD**	**ΔG_sol_**	**STD**	**ΔG_bind_**	
P53-MDMX	−287.56	5.00	16.91	1.06	241.52	4.25	−29.12	−336.96	5.58	18.87	1.20	290.65	4.42	−27.44	−9.12
P53-MDM2	−480.66	6.04	21.45	0.68	432.26	5.04	−26.95	−536.69	6.90	25.88	1.35	481.89	5.44	−28.93	−9.20
ΔΔG							2.17							−1.49	−0.08
PDIQ-MDMX	−201.31	4.18	11.82	0.68	163.05	3.27	−26.44	−237.69	4.65	13.14	0.73	196.33	3.72	−28.22	−9.5
PDIQ-MDM2	−372.41	4.81	14.47	0.69	320.47	3.49	−37.47	−402.49	5.39	19.66	1.60	351.71	4.30	−31.12	−11.0
ΔΔG							−11.03							−2.90	−1.50

*All values showed in the table in kcal/mol*.

* *The experimental value is obtained from Holak et al. (Popowicz et al., 2007, 2008) and Chen et al. (Phan et al., 2010)*.

**Table 2:** The energy terms under PPC-IE method for p53-MDMX/MDM2 and pDIQ-MDMX/MDM2 systems.

**Component**	**p53-MDMX**	**p53-MDM2**	**Delta**	**PDIQ-MDMX**	**PDIQ-MDM2**	**Delta**
Δ*E*_*vdW*_	−60.30	−79.65	−19.35	−61.92	−69.50	−7.58
Δ*E*_*ele*_	−276.66	−457.04	−180.38	−175.77	−332.99	−157.22
Δ*G*_*pol*_	298.71	491.58	192.87	204.06	359.84	155.78
Δ*G*_*nopol*_	−8.06	−9.69	−1.63	−7.73	−8.13	−0.40
−*TΔS*	18.87	25.88	7.01	13.14	19.66	6.52
Δ*G*_*ele*+*pol*_	22.05	24.85	2.80	28.29	26.85	−1.44
Δ*G*_*bind*_	−27.44	−28.93	−1.49	−28.22	−31.12	−2.90

*ΔE_vdW_, ΔE_ele_, ΔG_pol_, ΔG_nopol_, −TΔS, and ΔG_ele+pol_ represent van der Waals energy, electrostatic energy in the gas phase, polar salvation energy, non-polar solvation energy, entropy contribution, and the sum of the electrostatic energy with polar salvation energy, respectively. All values showed in the table are in kcal/mol*.

In addition, although the electrostatic interaction plays a highly beneficial role, most of its favorable factors are offset by the unfavorable polar solvation energy. Therefore, the favorable binding energies of p53-MDMX/MDM2 are mainly provided by vdW interactions. Furthermore, we analyze the differences in the electrostatic term, vdW term, polar solvation energy, non-polar solvation energy, and entropy change between p53-MDMX and p53-MDM2, which are −19.35, −180.38, 192.87, −1.63, and 7.01 kcal/mol, respectively. The stronger binding energy of p53-MDM2 compared with p53-MDMX is mainly due to the vdW interactions.

### The Analysis of Hydrogen Bond

To investigate the origin of the stronger binding affinity of p53 with MDM2 than with MDMX in detail, hydrogen bond analysis for p53-MDMX/MDM2 is performed and the hydrogen bond energy is calculated via the following formula (11) (Huang et al., 2008, 2012). The results are listed in Table 3.

(11) $$\begin{array}{l} \Delta G_{bind}=\frac{\alpha }{R^{12}}-\frac{\beta }{R^{10}} \end{array}$$

**Table 3:** Distance, occupancy, and energy of the hydrogen bonds formed from p53-MDMX/MDM2.

**System**	**Donor**	**Acceptor**	**Distance (Å)**	**Occupancy (%)**	**Energy (kcal/mol)**
p53-MDMX	LYS93-NZ-H	GLU110-OE1	2.75	99.94	−1.57
	TRP116-NE1-H	MET53-O	2.70	100.00	−2.87
	PHE112-N-H	GLN71-OE1	2.86	100.00	−1.31
p53-MDM2	TYR100-OH-HH	ASN122-O	2.58	100.00	−4.95
	TRP116-NE1-HE1	LEU54-O	2.78	100.00	−1.92
	PHE112-N-H	GLN72-OE1	2.96	100.00	−0.70
	ASN122-ND2-HD21	GLU25-OE1	2.90	100.00	−1.24

**Figure 2:**
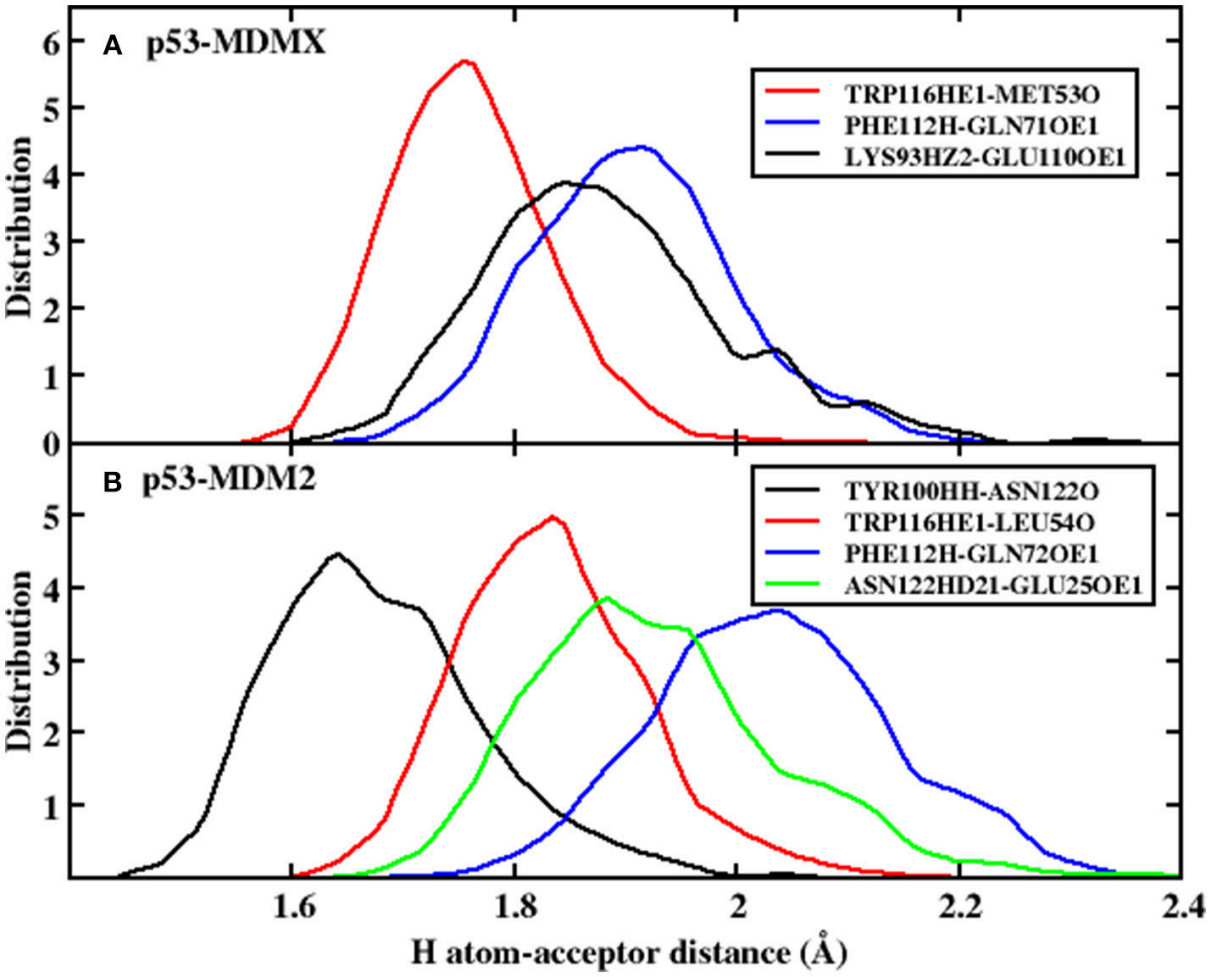
The frequency distribution of the distance for H atom-acceptor. **(A)** p53-MDMX system; **(B)** p53-MDM2 system.

**Figure 3:**
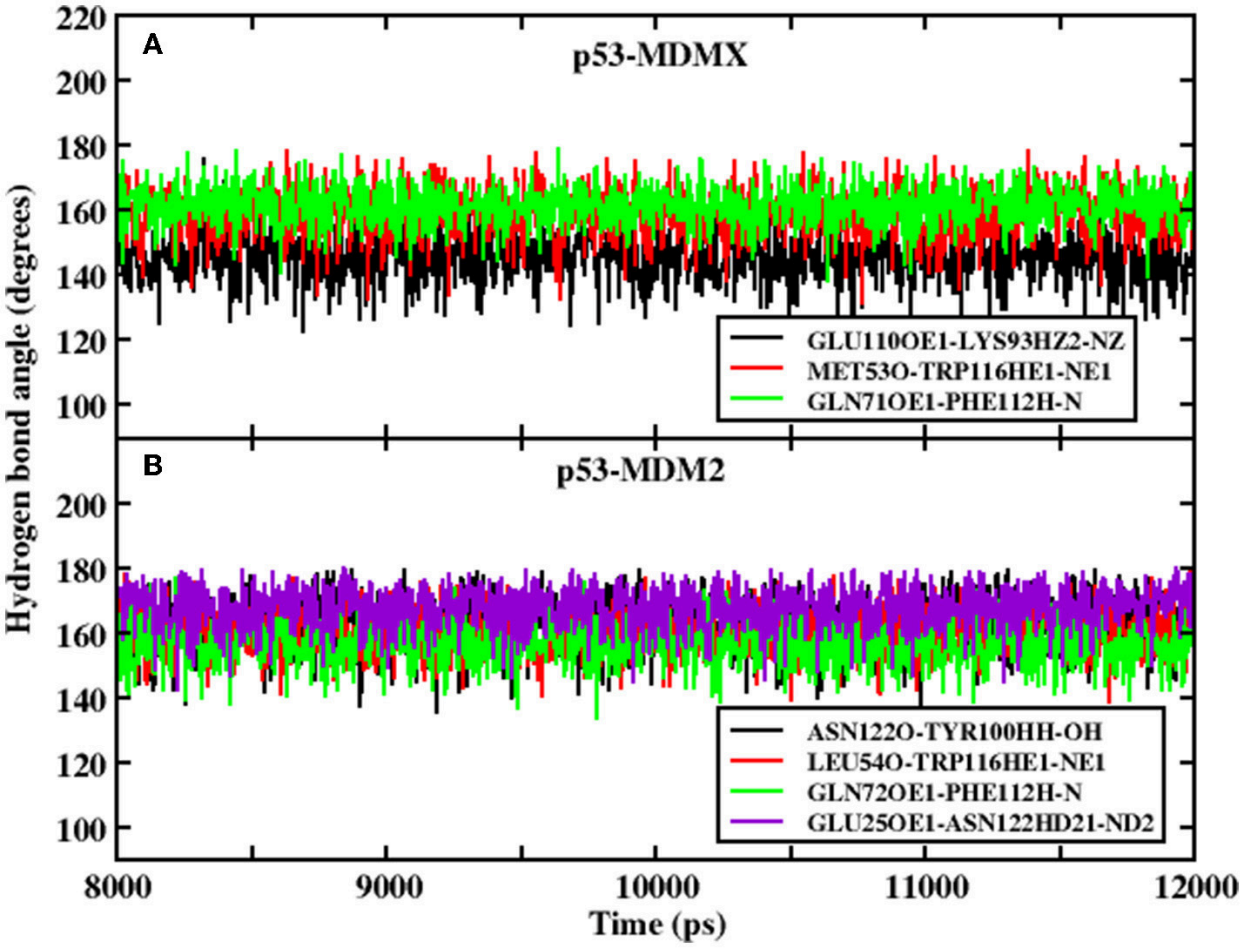
Evolution of the hydrogen bond angle between p53 and MDMX/MDM2 under PPC force field. **(A)** p53-MDMX system; **(B)** p53-MDM2 system.

In the equation, α and β have values 5.571 and 668.580, respectively, and *R* is the distance of the H-acceptor for the hydrogen bond. To obtain detailed information on the hydrogen bond, we plot the distance between the hydrogen atom and acceptor vs. their frequency distributions in Figure 2. There are three hydrogen bonds in the p53-MDMX system, while four hydrogen bonds are found in p53-MDM2. For the system of p53-MDMX, the peaks of the distance distributions for TRP116HE1-MET53O, PHE112H-GLN71OE1, and LYS93HZ2-GLU110OE1 are at ~1.7, 1.9, and 1.8 Å, respectively; hence, the formed hydrogen bonds are stable. For p53-MDM2, the peaks for TYR100HH-ASN122O, TRP116HE1-LEU54O, PHE112H-GLN72OE1, and ASN122HD21-GLU25OE1 are observed at 1.6, 1.8, 1.9, and 2.1 Å, respectively; hence, the formed hydrogen bonds are also highly stable. In addition, the time evolution of the hydrogen bond angle is shown in Figure 3. The angle remains >120 throughout the simulation time; therefore, the hydrogen bonds are well preserved under the PPC force field.

According to the above analysis, MDM2 can form more hydrogen bonds than MDMX with p53. Additionally, the total energy of the hydrogen bonds in p53-MDM2 is higher than in p53-MDMX, according to Table 3. This may explain the stronger binding affinity in p53-MDM2 than in p53-MDMX.

### The Decomposition of Residue

**Figure 4:**
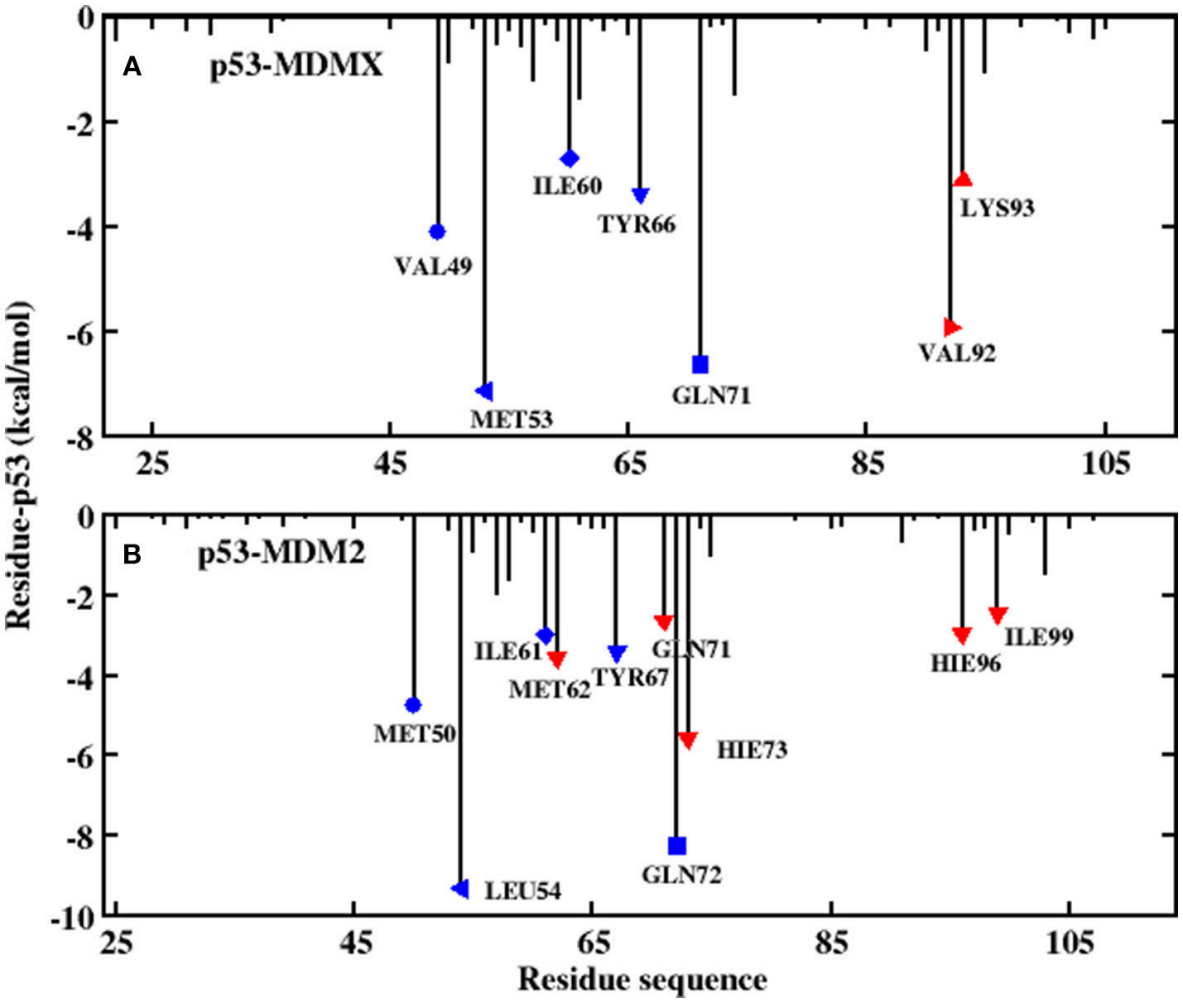
Decomposition of the binding free energy toward per-residue. **(A)** p53-MDMX system; **(B)** p53-MDM2 system.

**Figure 5:**
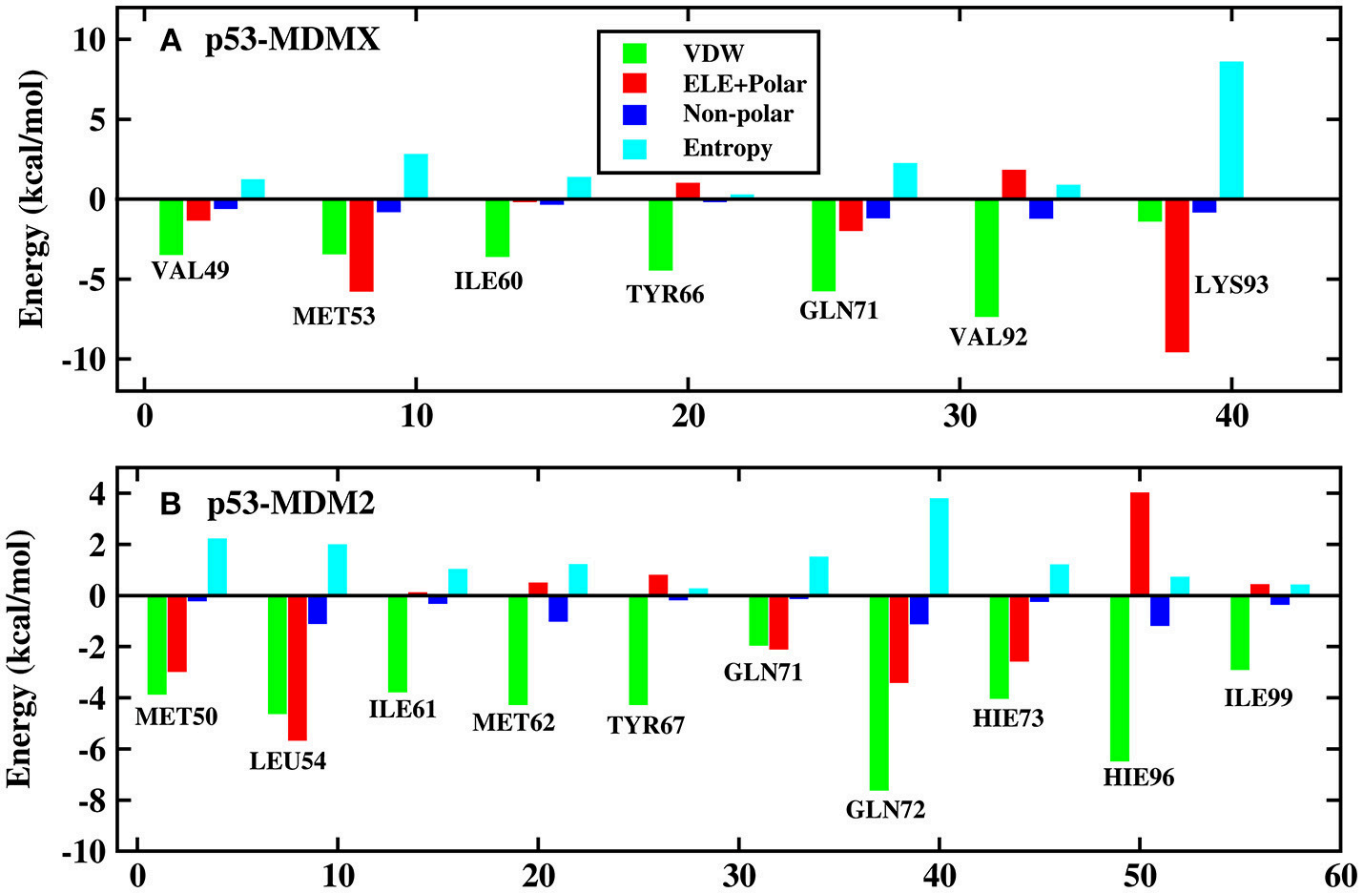
Decomposition of the binding free energy on a per-residue basis into contributions from electrostatic interactions, vdW energy, polar solvation energy, and non-polar solvation energy. **(A)** p53-MDMX system; **(B)** p53-MDM2 system.

To explore the reason for the decline in the effectiveness of p53 against MDM2 in residues, residue decomposition is conducted and the p53-residue interaction spectrum is depicted in Figure 4. To obtain more detailed information about the hot-spot residues, the binding free energy is divided into vdW interactions, the sum of the electrostatic energy and the polar solvation energy, the non-polar solvation energy, and the entropy change for the systems, which are shown in Figure 5.

In this work, the decomposition of residues is performed via MM/GBSA, combined with the IE method. According to Figure 4A, there are a total of seven residues with an energy contribution of more than −2 kcal/mol in the p53-MDMX system, including VAL49, MET53, ILE60, TYR66, GLN71, VAL92, and LYS93. According to Figure 5A, the vdW interactions and non-polar solvation energy contribute favorably to binding, while the entropy change plays an unfavorable role in every residue. For the hot-spot residues of VAL49, ILE60, TYR66, GLN71, and VAL92, the vdW interactions provide a dominant favorable contribution to the binding free energy, while the energy contributions from residues MET53 and LYS93 are dominated by electrostatic energy. For the p53-MDM2 system, as shown in Figure 4B, there are a total of 10 residues with an energy contribution of more than −2 kcal/mol and they are MET50, LEU54, ILE61, MET62, TYR67, GLN71, GLN72, HIE73, HIE96, and ILE99. According to Figure 5B, MET50, ILE61, MET62, TYR67, GLN72, HIE73, HIE96, and ILE99 are primarily driven by the vdW interactions for binding with p53, while the sum of the electrostatic energy and the polar solvation energy play the substantial role in the binding of LEU54/GLN71-p53.

**Figure 6:**
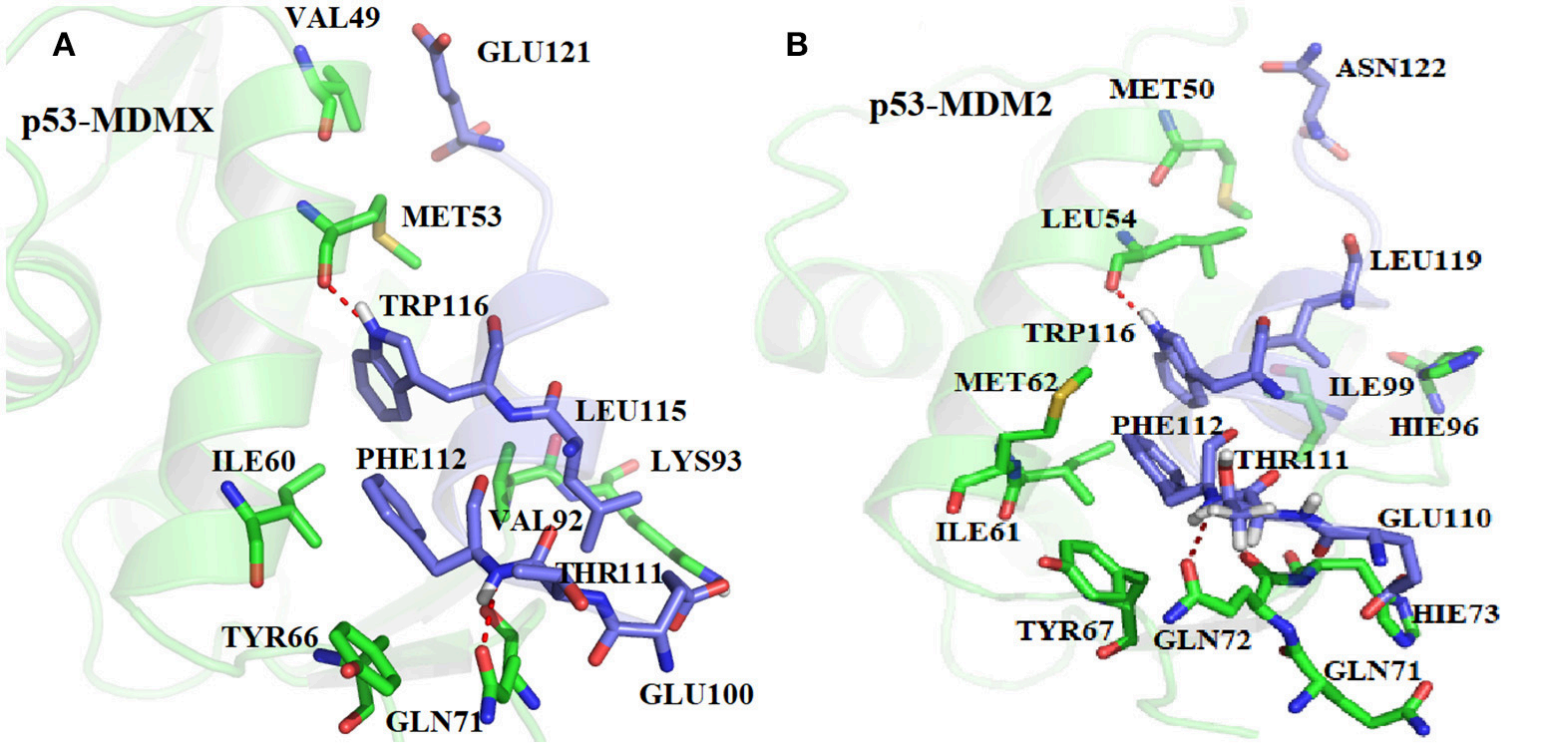
The relative position of the key residues. **(A)** p53-MDMX system; **(B)** p53-MDM2 system.

In order to get the specific explanation of the contribution for each hot-spot residue, the locations of the hot-spot residues are shown in Figure 6. As shown in Figure 6A, in the p53-MDMX system, the alkyl of residue VAL49 and the alkyl of residue GLU121 can form a CH-CH hydrophobic interaction. For residue ILE60, the most important contribution to the binding energy comes from the CH-π hydrophobic term between the alkyl of residue ILE60 and the phenyls of residue PHE112. In addition, the alkyl of ILE60 with the indole of TRP116 also forms a CH-π hydrophobic interaction. The phenyl of residue TYR66 is near the phenyl of residue PHE112; hence, a π-π hydrophobic interaction is formed. The oxygen atom of GLN71 can form a CH-O interaction with the CH group of THR111. The hydrogen bond that is formed between PHE112 and GLN71 also plays an important role in the binding. The length, occupancy and energy of this hydrogen bond are 2.86 Å, 100%, and −1.31 kcal/mol, respectively, as listed in Table 3; therefore, the hydrogen bond is very stable. Residue VAL92 forms a CH-CH hydrophobic interaction between the alkyl of residue VAL92 and the alkyl of residue LEU115. According to the above analysis of the hydrogen bond that is described in Table 3, MET53 and TRP116 form a hydrogen bond interaction with a hydrogen bond length, occupancy and energy of 2.70 Å, 100%, and −2.87 kcal/mol, respectively. Residue LYS93 forms a hydrogen bond with GLU110, of which the length is 2.75 Å, the occupancy is 99.94%, and the energy is −1.57 kcal/mol. In addition, a hydrophobic interaction is formed between the alkyl of residue LYS93 and the alkyl of residue LEU115, which also makes contributions.

According to, Figure 6B, the alkyl of MET50 and the alkyl of ASN122 form a CH-CH interaction. The CH group of ILE61 can interact with the indole of TRP116 and the benzene ring of PHE122. Residues MET62 and TYR67 are near residue PHE112; hence, it easy to produce a π-S interaction between MET62 and PHE112 and a π-π interaction between TYR67 and PHE112. The CH group of residue THR111 and the oxygen atom of GLN72 can form a CH-O interaction. Additionally, the oxygen atom of GLN72 forms a N-H group of residue PHE112, of which the distance, occupancy and energy are 2.96 Å, 100%, and −0.7 kcal/mol, respectively, as listed in Table 3. The binding free energy between HIE73 and p53 mainly comes from the CH-O interaction. The binding energy of HIE96 with p53 mainly comes from the CH-π interaction between the imidazole of HIE96 and the alkyl of LEU119. The binding contribution of ILE99 mainly comes from the CH-CH interaction between alkyls of ILE99 and LEU119. LEU54 has the strongest binding free energy with p53 of all residues and the main energy contribution comes from two parts, namely, the hydrogen bond interaction, which has an energy of −1.92 kcal/mol, and the CH-CH vdW interaction that is formed between LEU54 and LEU119, as shown in Figure 6B. The CH-O interaction from residues GLN71 and GLU110 contributes substantially to the binding free energy. Based on the above analyses, the CH-CH, CH-π, CH-O, and π-π interactions are the main forces that drive the binding between MDMX/MDM2 and p53.

### The Interaction Mechanism of pDIQ-MDMX/MDM2

Inhibiting efficiently the interaction of p53-MDMX/MDM2 can preserve the activation of p53, thereby preventing the occurrence of cancer. Therefore, in this paper, the interactions between an inhibitor, namely, pDIQ, and MDMX/MDM2 are also investigated to explore the binding mechanism.

The RMSD values of the backbone atoms relative to the native structure are plotted in Figure S1 in the Supporting Information and the time evolution of the interaction entropy is plotted in Figure 1. According to these figures, the simulation well converged; hence, the following analysis are reliable. The calculated binding free energy of pDIQ-MDMX/MDM2 using PPC-IE is listed in Table 1. The difference in the binding free energy between pDIQ-MDMX and pDIQ-MDM2 of −2.90 kcal/mol is consistent with the difference between the experimental values of −1.50 kcal/mol. Additionally, according to the PPC-IE results, pDIQ has a stronger binding affinity with MDMX/MDM2 than p53. The calculated binding free energies for pDIQ-MDMX/MDM2 are −28.22 and −31.12 kcal/mol, respectively, while they are −27.44 and −28.93 kcal/mol in p53-MDMX/MDM2. pDIQ also has a stronger binding affinity with MDM2 than with MDMX. These results well agree with the experimental results.

**Figure 7:**
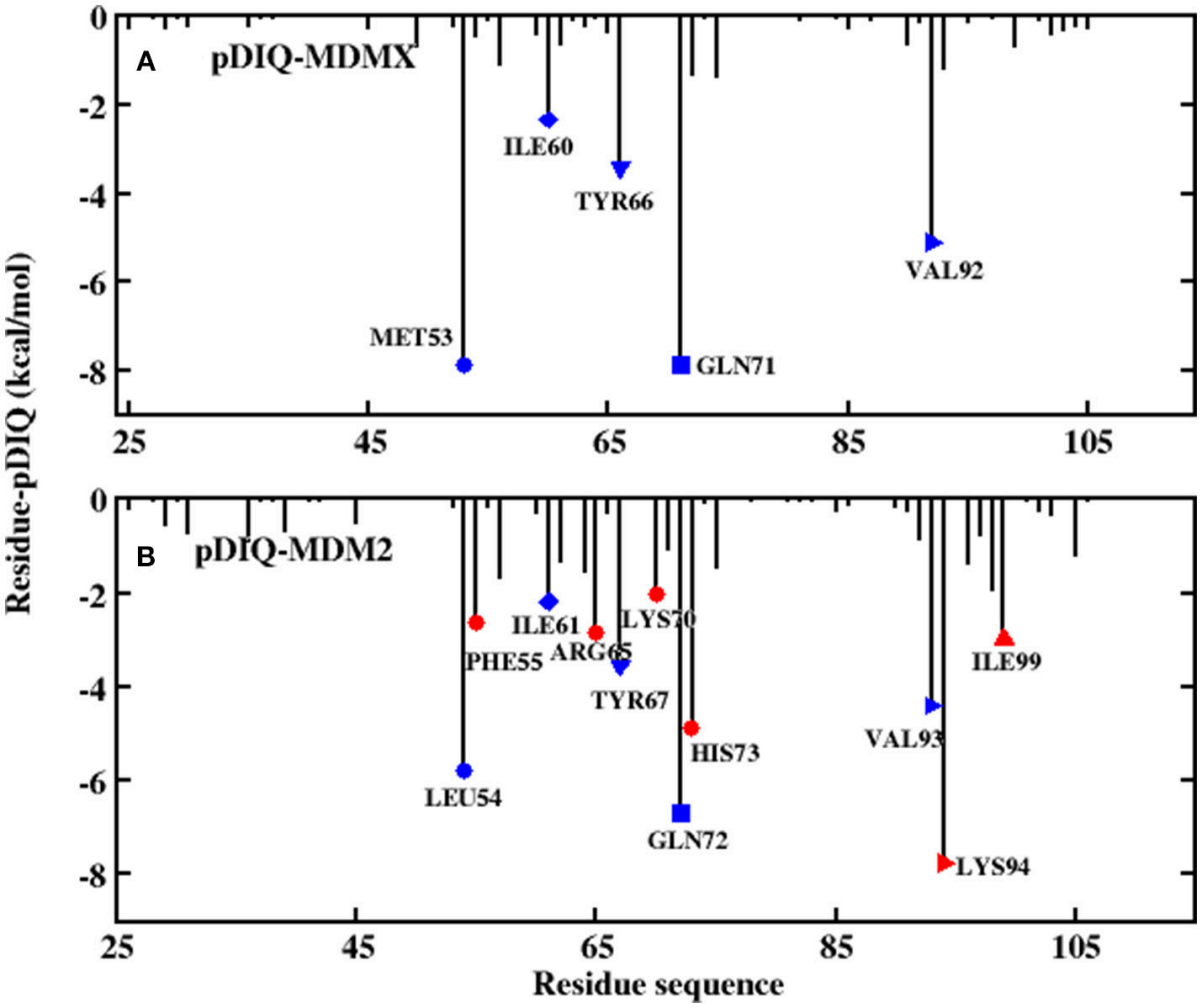
Decomposition of the binding free energy toward per-residue. **(A)** pDIQ-MDMX system; **(B)** pDIQ-MDM2 system.

Information on each energy component, including the electrostatic interactions, vdW interactions, non-polar solvation energies, polar solvation energy, and entropy change, for these two systems are listed in Table 2. Compared with pDIQ-MDMX, vdW interactions provide the major favorable contribution to the strong binding affinity in pDIQ-MDM2, which is consistent with the analysis of p53-MDMX/MDM2. To identify the contribution of every residue for the binding free energy to determine why pDIQ-MDM2 has higher binding ability than pDIQ-MDMX, the decomposition of residues is performed, the results of which are shown in Figure 7.

**Figure 8:**
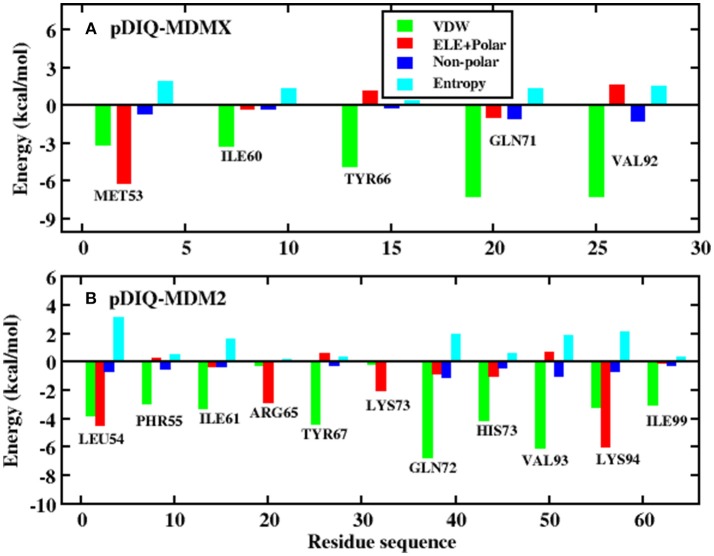
Decomposition of the binding free energy on a per-residue basis into contributions from electrostatic interactions, vdW energy, polar solvation energy, and non-polar solvation energy. **(A)** pDIQ-MDMX system; **(B)** pDIQ-MDM2 system.

According to Figure 5, there are five common-location key residues for MDMX and MDM2: MET53, ILE60, TYR66, GLN71, and VAL92 in MDMX and LEU54, ILE61, TYR67, GLN72, and VAL93 in MDM2. In the pDIQ-MDM2 system, there are additional hot-spot residues that are not present in the pDIQ-MDMX system: PHE55, ARG65, LYS70, HIS73, LYS94, and ILE 99. The total energy contribution of the hot-spot residues in pDIQ-MDMX is less than in pDIQ-MDM2. This may be one of the reasons why pDIQ with MDMX has a weaker binding affinity than pDIQ with MDM2. Moreover, the contributions of the binding free energy for hot-spot residues are decomposed into detailed terms for the two complexes, as shown in Figure 8. For pDIQ-MDMX, the energies of residues ILE60, TYR66, GLN71, and VAL92 are dominated by the vdW interactions, while that of MET53 is dominated by the electrostatic interactions. Similarly, for MDM2-pDIQ, the main energy contributions of the hot-spot residues come from vdW interactions, except for LEU54, ARG65, and LYS94, for which the electrostatic interactions dominate the energy contributions.

### The Analysis of p53 and pDIQ

**Figure 9:**
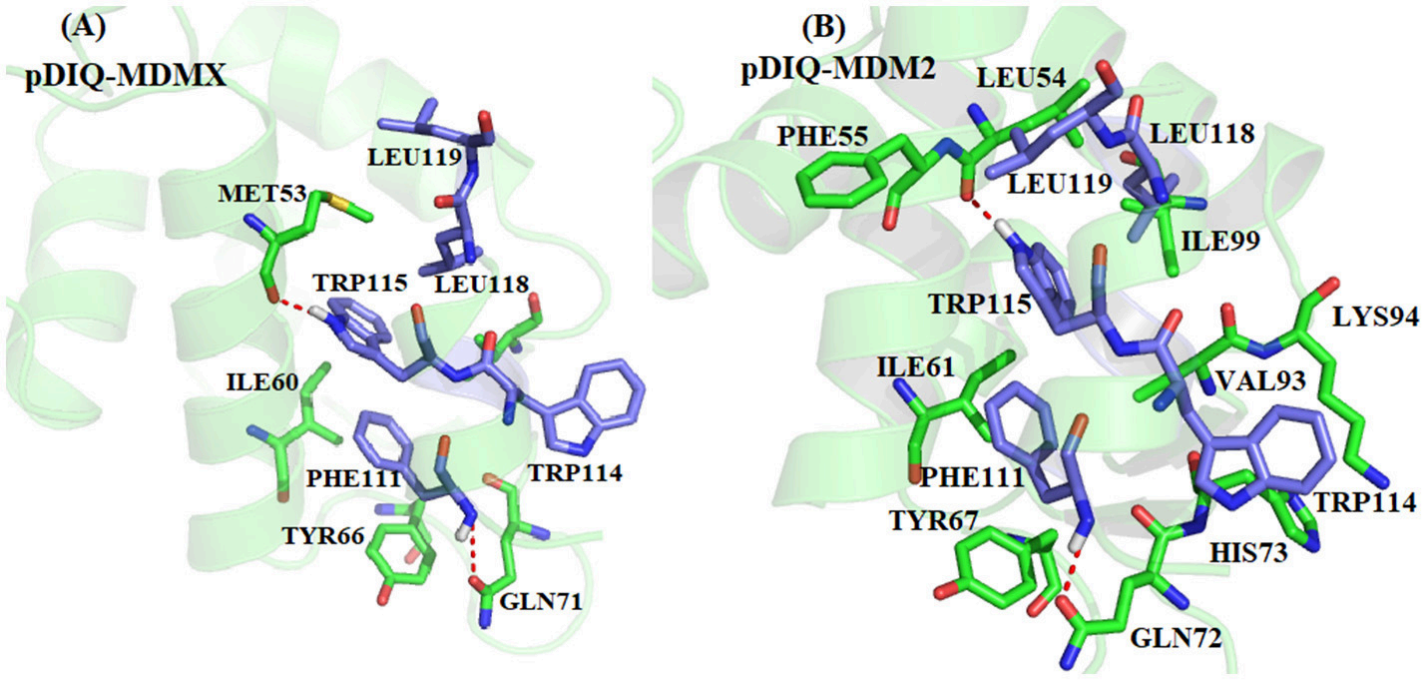
The relative position of the key residues. **(A)** pDIQ-MDMX system; **(B)** pDIQ-MDM2 system.

According to both the calculation results and the experimental measurements, pDIQ exhibits a stronger binding affinity with MDM2/MDMX than p53. According to Figures 4, 7, the hot-spot residues in p53-MDMX are almost identical to those in pDIQ-MDMX; they are MET53, ILE60, TYR66, GLN71, and VAL92. The positions of key residues relative to pDIQ are depicted in Figure 9. According to Figure 9A, the locations of the key spots for pDIQ are almost same as in the description of p53-MDMX in Figure 6. The binding modes of the key residues for p53-MDMX are similar to those for pDIQ-MDMX. However, the total residues contribution for pDIQ-MDMX is higher than for p53-MDMX, which results in a stronger binding affinity. For the p53-MDM2 and pDIQ-MDM2 systems, the main energy contribution comes from the six common residues: LEU54, ILE61, TYR67, GLN72, HIS73, and ILE99. They are also mainly dominated by the vdW interactions. Hot-spot residues that differ between the two systems are also identified: PHE55, ARG65, LYS70, VAL93, and LYS94 in pDIQ-MDM2 and MET50, MET62, GLN71, and HIE96 in p53-MDM2. The detailed interaction mechanism for each hot-spot residue with p53 has been explained in the previous analysis. Next, we analyze the hot-spot residues for pDIQ.

According to Figure 9, for PHE55-pDIQ, the energy contribution of −2.65 kcal/mol mainly comes from the CH-π interaction between the benzene ring of PHE55 and the alkyl of LEU119. The alkyl of VAL93 can generate interactions with the benzene ring of PHE111 and the indole of TRP 115, with a total energy contribution of −4.40 kcal/mol. For charged residue LYS 94, the binding free energy of −7.75 kcal/mol comes from the electrostatic interaction and the hydrophobic interaction between the alkyl of LYS93 and the indole of TRP114. For charged residues ARG65 and LYS70, the contributions to the binding energy are −2.86 and −2.02 kcal/mol, respectively, which mainly come from electrostatic interactions. The energy contributions of these hot-spot residues may lead to the difference in the binding affinity between p53-MDM2 and pDIQ-MDM2.

## Conclusions

In this paper, the non-polarized AMBER force field and the PPC force field, combined with the newly developed IE method, are used to explore the interaction mechanisms of p53 and 12mer peptide inhibitor pDIQ with MDMX/MDM2. The PPC force field considers the surroundings of the amino acids and accurately expresses the polarization effect. In addition, the IE method makes full use of all samplings that are obtained from the MD simulation, thereby providing accurate and effective results without increasing the computational cost. The binding free energy is calculated via the combination of MM/PBSA and the IE method under the AMBER and PPC force fields, respectively. Our results demonstrate the following:

First, the difference in the calculated binding free energy between p53-MDMX/MDM2 and pDIQ-MDMX/MDM2 is more consistent with the experimental difference that is observed under the PPC-IE method compared with AMBER-IE; hence, the electrostatic polarization effect plays an important role in the MD simulation. Moreover, the rank of the computed binding free energies are consistent with the rank of the experimental measurements under the PPC-IE method.

Second, according to the calculated binding free energy, the strength of MDM2 with p53 is stronger than that of MDMX with p53 because more hydrogen bonds are formed in p53-MDM2 than in p53-MDMX.

Third, according to the decomposition of the binding free energy, the vdW interactions are the main driving force for the binding in p53-MDMX/MDM2 and pDIQ-MDMX/MDM2. It is also the main cause of the weaker binding affinity of MDMX to p53/pDIQ compared with MDM2 to p53/pDIQ.

Fourth, pDIQ has stronger binding affinity with MDMX/MDM2 than p53 with MDMX/MDM2. The differences in the energy contributions of the hot-spot residues between the two systems lead to this phenomenon.

We hope this study can clarify the binding mechanisms of p53/pDIQ-MDMX/MDM2 and is helpful for designing a dual inhibitor that inhibits the p53-MDMX/MDM2 interaction.

## Author Contributions

ML and YC outperformed the MD simulations, drafted the main text of the manuscript, and prepared all the figures. YL, SZ, RW, and HL helped with data analysis. LD designed this study and revised the manuscript.

### Conflict of Interest Statement

The authors declare that the research was conducted in the absence of any commercial or financial relationships that could be construed as a potential conflict of interest.
