# Supplementary material for: Insight Into the Binding Mechanism of p53/pDIQ-MDMX/MDM2 With the Interaction Entropy Method
Source: Front Chem. 2019 Jan 29;7:33. doi: 10.3389/fchem.2019.00033 (PMC6361799; doi:10.3389/fchem.2019.00033)

# Supporting Information

# Insight into the binding mechanism of p53/pDIQ-MDMX/MDM2 with the interaction entropy method


Mengxin Li, [§,1] Yalong Cong, [§,1] Yuchen Li,[1] Susu Zhong,[1] Ran Wang,[1] Hao Li,[1,2], Lili Duan*[,1]

[1]School of Physics and Electronics, Shandong Normal University, Jinan 250014, China

[2]Department of Science and Technology, Shandong Normal University, Jinan 250014, China

Corresponding author (duanll@sdnu.edu.cn)

[§]Mengxin Li and Yalong Cong contributed equally to this work


**Figure S1** The RMSD of the protein backbone for four systems based on the AMBER force field and PPC force filed in the last equilibrium MD simulations. **(A)** p53-MDMX system; **(B)** p53-MDM2 system; **(C)** pDIQ-MDMX; **(D)** pDIQ-MDM2.

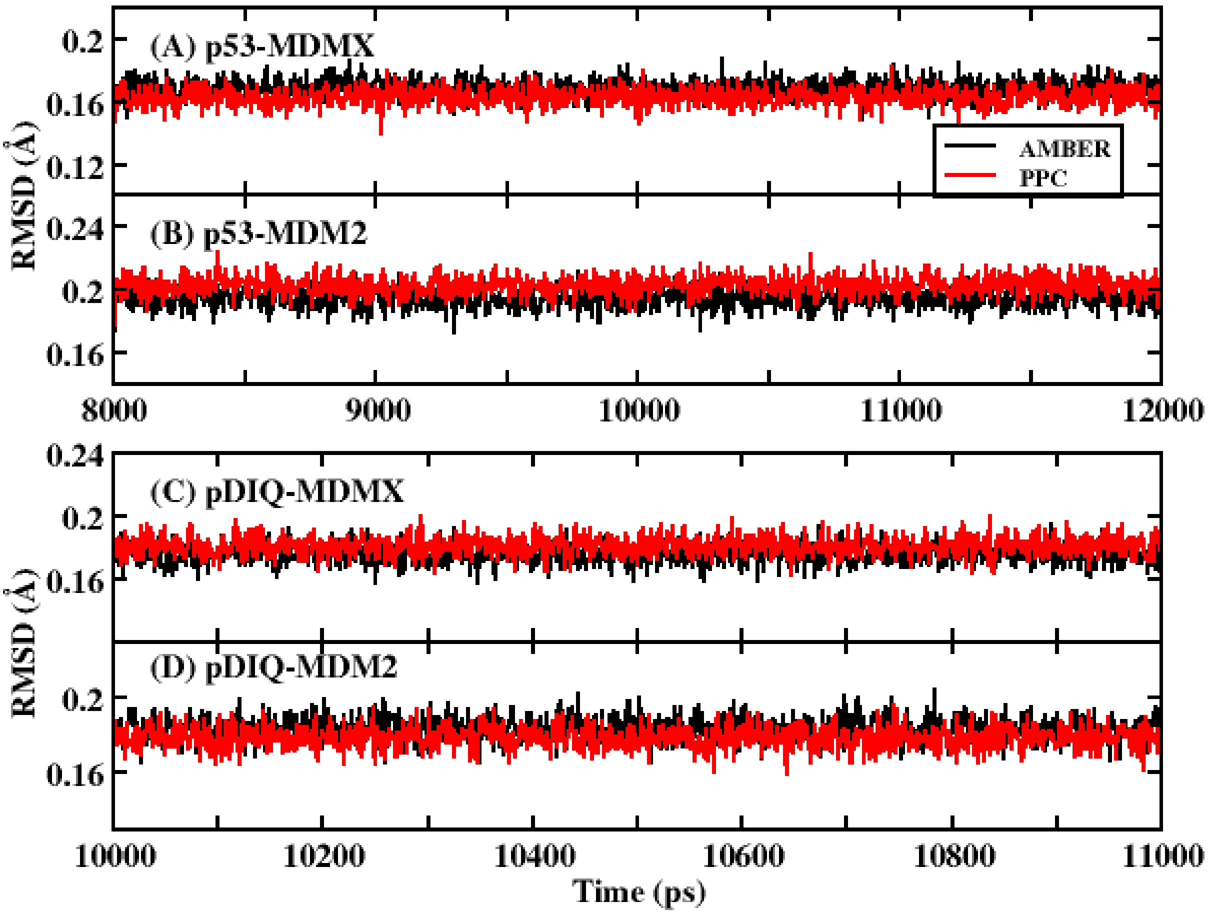

Supplement: Supplementary file 1 [file Data_Sheet_1.PDF]
